# Mental health, economic well-being and health care access amid the COVID-19 pandemic: a mixed methods study among urban men who have sex with men in India

**DOI:** 10.1080/26410397.2022.2144087

**Published:** 2022-12-07

**Authors:** Venkatesan Chakrapani, Peter A Newman, Aleena Sebastian, Shruta Rawat, Sandeep Mittal, Vanita Gupta, Manmeet Kaur

**Affiliations:** a Chairperson, Centre for Sexuality and Health Research and Policy (C-SHaRP), Chennai, India; DBT/Wellcome Trust India Alliance Senior Fellow, The Humsafar Trust, Mumbai, India. Correspondence: cvenkatesan@c-sharp.in, venkatesan.chakrapani@gmail.com; b Professor, Factor-Inwentash Faculty of Social Work, University of Toronto, Toronto, Canada; c Assistant Professor, National Institute of Advanced Studies (NIAS), Bangalore, India; d Research Manager, The Humsafar Trust, Mumbai, India; e Deputy Director (Targeted Interventions), Chandigarh State AIDS Control Society (CSACS), Chandigarh, India; f Project Director, Chandigarh State AIDS Control Society (CSACS), Chandigarh, India; g Professor, Postgraduate Institute of Medical Education and Research (PGIMER), Chandigarh, India

**Keywords:** MSM, COVID-19, depression, anxiety, social distancing, income security

## Abstract

Scant empirical research from Asia has addressed the impact of COVID-19 on sexual minority health. We aimed to explore and understand the impact of COVID-19 on income security, mental health, HIV risk and access to health services among men who have sex with men (MSM) in India. We conducted a concurrent mixed methods study from April to June 2020, including a cross-sectional survey and in-depth semi-structured interviews with MSM recruited from three non-governmental organisations providing HIV prevention services in Chandigarh, India. We examined the associations of sexual minority stressors (sexual stigma, internalised homonegativity), economic stressors, and stress due to social distancing, with depression and anxiety, HIV risk, and access to health services. Survey findings (*n* = 132) indicated that internalised homonegativity and stress related to social distancing were significantly associated with depressive and anxiety symptoms. Results also showed reduced access to condoms, HIV testing and counselling services. Qualitative findings (*n* = 10) highlighted adverse economic impacts of COVID-19, including loss of employment/wages and engaging in survival sex work, which contributed to psychological distress and HIV risk. The COVID-19 pandemic has resulted in considerable psychological and financial distress among low socioeconomic status MSM in India, including those involved in sex work – communities already marginalised in economic, family and healthcare sectors. Structural interventions to improve access to mental health and HIV services and decrease financial burden are critical to mitigate the impact of COVID-19.

## Résumé

Peu de recherches empiriques ont abordé l’impact de la COVID-19 sur la santé des minorités sexuelles. Nous souhaitions explorer et comprendre les répercussions de la COVID-19 sur la sécurité de revenu, la santé mentale, le risque de VIH et l’accès aux services de santé chez les hommes ayant des rapports sexuels avec des hommes (HSH) en Inde. D’avril à juin 2020, nous avons mené une étude concomitante à méthodes mixtes avec notamment une enquête transversale et des entretiens approfondis semi-structurés avec des HSH recrutés auprès de trois organisations non gouvernementales assurant des services de prévention du VIH à Chandigarh, Inde. Nous avons examiné les associations des facteurs de stress (stigmatisation sexuelle, homonégativité internalisée), des facteurs de stress économiques et des tensions dues à la distanciation sociale avec la dépression et l’anxiété, le risque de contracter le VIH et l’accès aux services de santé. Les résultats de l’enquête (*n* = 132) ont indiqué que l’homonégativité internalisée et les tensions relatives à la distanciation sociale étaient associées de façon non négligeable à des symptômes de dépression et d’anxiété. Les résultats ont aussi révélé un accès réduit aux préservatifs, ainsi qu’aux services de dépistage du VIH et de conseil. Les résultats qualitatifs (*n* = 10) ont mis en lumière les conséquences économiques négatives de la COVID-19, notamment la perte de l’emploi/du salaire et le recours au commerce du sexe pour survivre, ce qui a contribué à une détresse psychologique et un risque de VIH. La pandémie de COVID-19 a causé une détresse psychologique et financière considérable chez les HSH à faible statut socioéconomique en Inde, y compris ceux qui participent au commerce du sexe – des communautés déjà marginalisées dans les secteurs économiques, familiaux et sanitaires. Des interventions structurelles pour élargir l’accès aux services de santé mentale et de lutte contre le VIH de même que pour alléger la charge financière sont capitales pour atténuer l’impact de la COVID-19.

## Resumen

Pocas investigaciones empíricas realizadas en Asia han abordado el impacto de COVID-19 en la salud de minorías sexuales. Procuramos explorar y entender el impacto de COVID-19 en la seguridad de ingresos, la salud mental, el riesgo de VIH y el acceso a servicios de salud entre hombres que tienen sexo con hombres (HSH) en India. Realizamos un estudio de métodos mixtos concurrente entre abril y junio de 2020, que incluía una encuesta transversal y entrevistas semiestructuradas a profundidad con HSH reclutados de tres organizaciones no gubernamentales que proporcionan servicios de prevención del VIH en Chandigarh, India. Examinamos las asociaciones de los estresores de minorías sexuales (estigma sexual, homonegatividad internalizada), estresores económicos y estrés debido al distanciamiento social, con depresión y ansiedad, riesgo de VIH y acceso a servicios de salud. Los hallazgos de la encuesta (*n* = 132) indicaron que la homonegatividad internalizada y el estrés relacionado con el distanciamiento social estaban asociados de manera significativa con síntomas de depresión y ansiedad. Además, los resultados mostraron menor acceso a condones, pruebas de VIH y servicios de consejería. Los hallazgos cualitativos (*n* = 10) destacaron impactos económicos adversos de COVID-19, tales como pérdida de empleo/sueldo y participación en trabajo sexual de supervivencia, que contribuía a angustia psicológica y al riesgo de VIH. La pandemia de COVID-19 ha causado considerable angustia psicológica y financiera entre HSH de bajo nivel socioeconómico en India, incluidos aquéllos involucrados en trabajo sexual, comunidades ya marginadas en sectores económicos, familiares y sanitarios. Las intervenciones estructurales para mejorar el acceso a servicios de salud mental y VIH y disminuir la carga financiera son esenciales para mitigar el impacto de COVID-19.

## Introduction

Globally, COVID-19 continues to impact the lives of all communities, including gay and other men who have sex with men (MSM). As of 11 September 2022, India ranked second globally in the number of SARS-CoV-2 infections,^1^ with 528,057 deaths reported due to COVID-19.^2^ Several studies from the United States and other countries have documented negative impacts of COVID-19 on the mental health and economic well-being of sexual and gender minorities.^3–5^ However, there is limited empirical data on the impact of the pandemic on MSM communities in India. Pervasive barriers in access to health care services and challenges in engaging in safer sex practices have been documented among MSM before the onset of the COVID-19 pandemic.^6–8^ Socioeconomic and psychological stressors experienced during the pandemic and associated lockdowns may have exacerbated service disruptions and psychological distress among MSM.

Minority stress theory and syndemic theory offer nuanced perspectives for understanding the interconnections between sexual identity, mental health and HIV risk.^9,10^ Studies from India have shown how stigma and discrimination are linked to the psychological and sexual health of MSM.^11,12^ According to minority stress theory, social-structural factors such as discrimination and marginalisation of sexual minorities negatively impact mental health.^13^ Syndemic theory complements the minority stress model by postulating that social-structural conditions like stigma and discrimination contribute to the co-occurrence of two or more psychological health conditions that may act synergistically to increase adverse health outcomes, including HIV risk.^14,15^

Consistent with minority stress theory^14^ and syndemic theory,^16^ empirical studies have shown that stigma, discrimination, and other forms of marginalisation faced by MSM in India contribute to synergistic epidemics (or syndemics) of psychosocial health conditions, such as depression^17^ and internalised homonegativity,^9,15^ which increase HIV risk.^18,19^ For example, fatalistic attitudes and alcohol use can lead to condomless sex and thus increase the risk of contracting or transmitting HIV.^12,20,21^ In fact, studies have shown a high prevalence of HIV among MSM (2.7–13.1%) in India, which could partly be explained by syndemic theory.^22–24^ Stigma and discrimination due to same-sex sexuality have been shown to impede access to HIV and sexual health services.^25–29^ In the context of COVID-19, economic stressors^30^ and stress due to social distancing may increase psychological distress among MSM. Decreased access to condoms, HIV and STI testing and treatment due to COVID-19-related restrictions (e.g. closure of HIV prevention services) may further contribute to HIV risk.^5^

Guided by minority stress and syndemic theories, we aimed to understand the effects of sexual minority stressors (such as the experience of sexual stigma and internalised homonegativity), and COVID-19-related socioeconomic and social distancing stressors on psychological distress and access to healthcare services. We hypothesised that both types of stressors (sexual minority and COVID-19) would increase the risk of psychological distress and subsequently HIV risk among MSM.

## Methods

From April to June 2020, following the first nationwide pandemic lockdown in March 2020, we conducted a concurrent mixed methods study with MSM in Chandigarh (population 1.1 million), the capital city of two northern Indian states. Interviews were conducted in person, following COVID-19 precautionary measures recommended by the World Health Organization:^31^ participants and interviewers wore face masks and gloves, used hand sanitizers, and maintained physical distancing. Disposable masks and gloves and hand sanitizers were provided to all participants.

### Quantitative

A convenience sample of MSM was recruited from three non-governmental organisations (NGOs) providing HIV prevention services in Chandigarh. Eligible participants were at least 18 years old and self-identified as MSM, including a diversity of sexual self-identifications (e.g. gay, bisexual, *kothi* – usually feminine and receptive role, *panthi* – insertive role, *double-decker* – insertive and receptive role).^9^ We conducted a cross-sectional, interviewer-administered survey in the Hindi language. This study was part of an intervention to improve HIV testing and mental health among MSM in Chandigarh, and the protocol was approved by the ethics committee of the Postgraduate Institute of Medical Education and Research (IEC-11/2015-293, dated 28 August 2019). For the intervention study, the calculated sample size was 232 (anticipated effect size = 0.25, power = 80, alpha = 0.05, using GPower 3.1). As the COVID-19-related questions were added to the post-intervention assessment tool, there was no formal sample size estimation for the survey on the impact of COVID-19 among a subset of intervention participants. All participants provided written informed consent.

Survey items included sociodemographic characteristics, sexual minority, economic, and COVID-19-specific stressors, mental health, access to HIV/sexual health and mental health services, and COVID-19 protective behaviours during sex. The entire questionnaire, including the scales, was translated from English to Hindi by professional translators. The questionnaire was pilot-tested with 10 MSM to ensure that the translated scale items were relevant and comprehensible (content validity).

#### Sociodemographic characteristics

We collected information on age, education, occupation, income, marital status, sex work status, HIV status and sexual identification.

#### Sexual minority stressors

Sexual stigma (experience of stigma and discrimination due to one’s sexual minority identity) was assessed using a 15-item MSM stigma scale that has been validated in South India.^18^ This scale was used to assess perceived and enacted sexual stigma. Perceived stigma examines a person’s knowledge of the prevalent stigma and discrimination towards sexual minority communities in general and the risk of oneself being exposed to such experiences because of one’s minority status. Enacted sexual stigma explains one’s overt negative experiences due to sexual minority identity. These negative experiences include verbal abuse, bullying, physical harassment, sexual abuse, loss of peer circle and loss of career opportunities. Cronbach’s alpha for the sexual stigma scale in this study was 0.70.

Internalised homonegativity (internalisation of society’s negative attitudes towards same-sex sexuality) was assessed using five items from Herek’s revised internalised homophobia scale^32,33^ that has been tested in India as well.^18^ The revised scale had five statements: (1) you tried to stop being attracted to men in general, (2) if someone had offered you the chance to be completely heterosexual, you would have accepted the chance, (3) you wished you were not attracted to men, (4) you felt that being [MSM] was a personal shortcoming for you, and (5) you wanted to get professional (e.g. psychiatrist) help in order to change sexual orientation to heterosexual. The responses ranged from “Strongly Disagree” (1) to “Strongly Agree” (5). Cronbach’s alpha for this scale in this study was 0.97.

#### Economic stressors

Economic stress was assessed by the questions, “Since the initiation of the first nationwide lockdown until now, in order to cover daily expenses, have you: taken loans (yes/no), sold assets (yes/no), pawned things or land (yes/no)”; and “Do you have outstanding loans from before lockdown? (yes/no)”. Participants who answered “yes” to any of these items were coded as “yes” for economic stressors.

#### COVID-19-specific stressor

Stress due to social/physical distancing was assessed by a single item: “Have recommendations for social distancing caused stress for you?” (not at all = 0, a little  = 1, somewhat = 2, a lot = 4).

#### Mental health

Experience of depressive symptoms since lockdown (past 1–3 months) was assessed with a single item based on Patient Health Questionnaire-2 (PHQ-2)^34^: “During the lockdown, did you feel down, depressed or hopeless?” Similarly, the experience of anxiety symptoms since lockdown was measured using a single item based on Generalized Anxiety Disorder-2 (GAD-2)^35^: “During the lockdown, did you feel nervous, anxious or on edge?” As PHQ-2 and GAD-2 measured symptoms in the past two weeks, we did not use the scores from these scales, and used only the first item from each of these scales as we were interested in assessing the cumulative impact of the lockdown on mental health.

#### Access to HIV/sexual and mental health services

Access to condoms, HIV testing, STI testing/treatment and mental health counselling were assessed as follows:

“Since the beginning of lockdown (March 24, 2021) until now, could you: 1) get condoms when needed? 2) get tested for HIV? 3) see a doctor for screening or treatment of sexually transmitted diseases? and 4) get mental health counselling?”

Response options were yes, no, and not applicable.

#### Protective behaviours

Participants were asked whether they used each of the following during post-lockdown sexual encounters: condoms, COVID-protective masks, gloves, and hand sanitizer (yes/no).

### Qualitative

We conducted in-depth interviews with a purposive sample of MSM recruited from the three NGOs using maximum variation sampling to ensure diversity by sexual identity (i.e. gay, *kothi*, and so on), sex work and HIV status. After obtaining written informed consent, interviews were conducted in Hindi by trained peer interviewers. The interviews were audio-recorded, transcribed and translated into English. An in-depth interview topic guide was used to explore participants’ experiences during the lockdown, including the impact of the lockdown on income, mental health, sexual life and access to health services, especially HIV prevention and treatment services. Details of some of the topics covered in the topic guide are provided below.

#### Access to HIV-related services

Participants’ experiences in accessing HIV-related services were explored through questions such as: “After the initiation of lockdown until it is revoked, could you contact outreach workers or peer educators of the NGOs that provide HIV services? Why or Why not?” and

“Whether you required any HIV services (such as condoms, lubes, HIV testing, screening and treatment of sexually transmitted diseases, mental health counselling and HIV medications) during the lockdown period? If yes, did you get those services? If you got those services, please provide details on how you got those services (who, where, which agency, etc.).”

#### Mental health

Psychological distress was assessed by questions and probes such as:

“During the lockdown, what kind of problems have you faced? Have you faced any issues that affected your mental health? Can you elaborate on those issues? (e.g. forced to stay with unsupportive family members; violence from family, partner or police); What kind of impact have those issues had on your mental health?”

and “To what extent were your mental health concerns related to your job or financial situation? Can you elaborate on those aspects?”

#### Sexual life

This was explored by asking questions such as: “During the lockdown, did you meet any male sexual partners (casual, paying or paid partners)?” and “Were you able to use condoms? Why or why not?” Participants were also asked whether they used masks, sanitizers and gloves during the sexual encounter, and (if not) their reasons for not using COVID-protective safety measures.

#### Livelihood and support

Among those who were unemployed, we probed whether they were unemployed before lockdown or lost their job after the onset of lockdown. Those who reported financial distress during lockdown were asked: “Can you tell us how you managed to earn or support yourself and your family during the lockdown?” and “Did you receive support (monetary or in-kind) from your family members, relatives, friends, NGOs or others during the lockdown, and now?” Participants were also asked whether they obtained loans during the lockdown. We also asked about the nature of support (e.g. emotional, material or monetary) received from peers, CBOs and government agencies.

### Data analysis

#### Survey

Descriptive statistics were used to summarise categorical and continuous variables. We conducted multivariate logistic regression analyses to identify correlates of depression and anxiety by including sexual minority stressors and COVID-19-specific stressors as independent variables. There were no missing data for the variables included in the regression models. The control variables included were: age (0 = <25 years, 1 = ≥25 years); education (0 = Completed up to high school, 1 = Completed higher secondary school and above); marital status (0 = Currently single, 1 = Currently married to a woman); engagement in sex work (0 = Not in sex work, 1 = In sex work); HIV status (0 = HIV-negative/unknown status, 1 = HIV positive); and sexual or sexual role-based identity (0 = *Kothi*, 1 = Double-Decker/Versatile, 2 = Top/*Panthi*/Gay/Bisexual).

#### Qualitative component

A bilingual Hindi-English translator listened to the Hindi recordings and translated them into English. Next, two independent researchers conducted thematic analysis using techniques (e.g. open coding, constant comparison) adapted from the grounded theory approach.^36^ Qualitative findings complemented quantitative findings and helped to explain the associations found in quantitative analyses.^37^

## Results

### Sociodemographic and related characteristics

The mean age of survey participants (*n* = 132) was 27 years and mean monthly income was INR 8375 (US$ 120) (Table 1). A majority (79%) were unmarried and 61% identified as *Kothi.* Nearly two-thirds (64%) were college graduates. One-fourth (*n* = 34; 26%) were unemployed, while others were private company staff (37%, *n* = 49), daily wage labourers (6%, *n* = 8), self-employed (5%, *n* = 7), staff of community-based organisations (3%, *n* = 4), government employee (1%, *n* = 1) or engaged in sex work (17%, *n* = 22). Seven MSM (5%) were students. Overall, 43% (*n* = 57) engaged in sex work, 26% (*n* = 34) were unemployed, and 8% (*n* = 10) reported being HIV positive.

**Table 1.** Sociodemographic characteristics of study participants (*n* = 142)

Characteristics	Survey participants (*n* = 132) Mean (SD) or *n* (%)	Qualitative in-depth interview participants (*n* = 142) Mean (SD) or *n* (%)
Age in years	27.2 (4.2)	27.7 (4.5)
Personal monthly income (INR)	8375 [US$ 120] (6847)	9778 [US$ 135] (5473)
Marital status		
Unmarried	104 (78.8)	7
Married	28 (21.2)	3
Education		
Higher secondary or lower	47 (35.6)	5
Graduate degree or higher	85 (64.4)	5
Employment		
Private company staff	49 (37.1)	2
Sex worker	22 (16.7)	3
Unemployed	34 (25.8)	4
Daily wage labourer	8 (6.1)	1
Self-employed	7 (5.3)	
Student	7 (5.3)	
Staff of community-based organisations	4 (3.0)	
Government employee	1 (0.8)	
Engagement in sex work in the past 6 months		
No	75 (56.8)	7
Yes	57 (43.2)	3
Sexual identification		
*Kothi* or Bottom	80 (60.6)	5
*Double-Decker* or Versatile	34 (25.7)	2
Gay	11 (8.3)	3
*Panthi or* Top	3 (2.3)	
Bisexual	4 (3.0)	
HIV status		
Positive	10 (7.6)	2
Negative	122 (92.4)	8

Participants (*n* = 10) in qualitative in-depth interviews had a mean age of 28 years and a mean monthly income of INR 9778 (US$ 135). Seven MSM were unmarried and five had a college degree. Two MSM reported being HIV positive. Four were unemployed, three reported engaging in sex work, two MSM were private company staff, and one was a daily wage labourer (Table 2). Five participants self-identified as *kothi* or bottom, three as gay and two as double-decker or versatile.

**Table 2.** Survey component: key variables such as stressors, psychological distress and access to health services (*n* = 132 participants)

Stressor	Survey participants (*n* = 132) Mean (SD) or *n* (%)
Sexual stigma total score	18.6 (3.4)
Internalised homonegativity total score	9.1 (3.8)
*Social distancing-related stress*	
Have recommendations for social distancing caused stress for you?	
Not at all	36 (27.3)
A little	33 (25.0)
Somewhat	51 (38.7)
A lot	12 (9.0)
*Psychological distress*	
*Depressive symptoms*	
During the lockdown, did you feel down, depressed, or hopeless?	
No	17 (12.9)
Yes	115 (87.1)
*Anxiety symptoms*	
During the lockdown, did you feel nervous, anxious or on edge?	
No	42 (31.8)
Yes	90 (68.2)
*Access to health services*	
*Access to condoms* (*n* = 128)	
No	25 (19.5)
Yes	103 (80.5)
*Access to HIV testing* (*n* = 124)	
No	47 (37.9)
Yes	77 (62.1)
*Access to screening or treatment of STIs* (*n* = 119)	
No	65 (54.6)
Yes	54 (45.4)
*Access to mental health services* (*n* = 65)	
No	48 (73.8)
Yes	17 (26.2)

### Economic impact

Among survey participants, 26% (*n* = 34) reported having procured loans during the lockdown, and 23% (*n* = 30) had outstanding loans from before the lockdown. Among those who reported having currently outstanding loans (*n* = 56), the majority (55%; *n* = 31) reported that they did not have the funds to pay, 35% (*n* = 20) said that they could pay instalments on time, and 9% (*n* = 5) reported having received an extension for loan payment.

In the in-depth interviews, job layoff was reported by four MSM working in private companies. One participant said, *“I used to work in a club. During the lockdown, I lost my job and my salary stopped”* (IDI-5, versatile, age 28, single). Financial stress was experienced by a few participants through delays in salary as reported by one of them: *“I am a make-up artist in a salon. I have not received two months of salary”* (IDI-3, gay, age 24, single).

For those who were self-employed or engaged in informal labour, reduced income also created an economic burden. According to a participant, *“I earn my daily wage as a carpenter. Due to lockdown, I am unable to continue my work and my income has been substantially reduced”* (IDI-7, *kothi*, age 35, married and living with family, HIV positive).

Loss of employment was identified as a stressor by participants, who described trying to find alternative sources of income. Among four MSM who reported job layoffs during COVID-19, two of them chose to engage in sex work due to economic necessity. One of those who engaged in sex work said:

*“I lost my private company job during the lockdown. There were no savings left and I had to choose sex work to earn a living. But I received fewer clients in person as they were afraid of getting Coronavirus infection. I then found a client through [a gay dating app]. But he refused to pay me after having sex. I did not share this information with anyone.”* (IDI-10, bottom, age 33, married, living with HIV)

As the above narrative indicates, those who engaged in sex work during the COVID-19 pandemic had a lower number of clients and some were cheated by their clients who refused to pay after receiving services. It is possible that as some participants entered into sex work only after the onset of the COVID-19 pandemic, and under economic pressure, they lacked negotiation skills and did not anticipate that some clients might not pay, and also were in less of a position to refuse potential clients.

Due to salary delays, cuts, or loss of wages, three MSM reported struggling to buy food and cooking gas, and to pay electric bills. A sex worker reported borrowing money (through mobile payment apps) from a regular client by promising sex later. A participant living with HIV and on antiretroviral treatment reported that economic hardship led to challenges in accessing nutritious food.

### Mental health impact

Over two-thirds (68%) of survey participants reported that they “felt nervous, anxious, or on edge” (categorised as having “anxiety symptoms”), and 87% reported that they “felt down, depressed, or hopeless” (“depressive symptoms”) during the lockdown. Nearly three-quarters (73%) reported experiencing stress due to social distancing restrictions. In logistic regression analyses, stress due to social distancing was significantly associated with depressive (aOR = 6.33, 95% CI 2.06, 19.45, *p* = .001) and anxiety symptoms (aOR = 5.02, 95% CI 2.37, 10.65, *p* < .001) (Table 3). Similarly, internalised homonegativity was significantly associated with both depressive (aOR = 1.65, 95% CI 1.22, 2.23, *p* = .001) and anxiety symptoms (aOR = 1.41, 95% CI 1.11, 1.77, *p* = .004).

**Table 3.** Predictors of depression and anxiety since lockdown: multivariable logistic regression analyses

Variables	Depression			Anxiety		
	aOR	SE	95% CI	aOR	SE	95% CI
Predictors						
*Sexual minority stressors*						
Internalised homonegativity score	1.65**	0.25	1.22–2.23	1.41**	0.17	1.11–1.77
Sexual stigma score	1.05	0.11	0.85–1.29	1.04	0.12	0.83–1.31
Economic stress (Yes)	1.89	1.58	0.37–9.73	3.61*	2.32	1.03–12.69
Stress due to social distancing (0–3)	6.33**	3.63	2.06–19.45	5.02***	1.93	2.37–10.65
*Control variables*						
Completed higher secondary school and above (Ref. Up to high school)	3.09	2.89	0.49–19.39	3.01	1.96	0.84–10.78
Age (≥25 years)	1.30	1.10	0.25–6.80	1.08	0.58	0.38–3.07
Sex work (Yes)	3.79	2.87	0.86–16.77	0.26*	0.16	0.08–0.89
Married (Ref. Single)	3.19	2.90	0.54–18.93	3.29	2.68	0.67–16.19
HIV positive (Ref. HIV-negative)	–	–	–	5.07	6.56	0.40–63.89
*Sexual identity* (Ref. *Kothi*)						
Double-Decker/Versatile	0.06*	0.07	0.01–0.60	0.39	0.34	0.07–2.20
Top/*Panthi*/Gay/Bisexual	0.03**	0.03	0.00–0.25	0.10**	0.08	0.02–0.53

Note: aOR = Adjusted Odds Ratio, SE = Standard Error, CI = Confidence Interval.

*p < .05, **p < .01, ***p < .001.

Qualitative findings indicated that those who resided alone had no face-to-face contact with their peers and friends. According to a participant in sex work:

*“What lies in the future is worrisome. There is not enough money left and no one to talk to. During the lockdown, I mostly spent days in my room and clients were visiting me. I could not share my personal problems with them. As I was unable to meet my [MSM] friends, I felt lonely and depressed.”* (IDI-8, *kothi*, age 25, single, sex worker)

Although many MSM reported using social media (e.g. Facebook), online messengers (e.g. WhatsApp), and gay dating apps (e.g. Grindr, Blued), many of those who lived alone reported depressive and anxiety symptoms. An MSM living with HIV reported suicidal ideation due to anxiety about not earning sufficient income to support his family:

*“I am married. My job was contract-based, and I was laid off during lockdown. As I am on [HIV treatment], I need to consume sufficient food. But there is no money, and I am scared thinking of how to live going forward. I thought of committing suicide many times. But I controlled myself when I thought of my wife, daughter and elderly parents who are dependent on me.”* (IDI-10, bottom, age 33, married, staying alone, HIV positive)

MSM who stayed with family members reported not meeting with peers and limited phone time with peers due to fear of accidental disclosure of their sexual identity or HIV status to family members, with potentially negative consequences such as violence and eviction. One participant reported that his friend was evicted from home after his family discovered his sexuality. Two other participants reported that their parents stopped talking to them after they disclosed their sexuality, and as a result, they received no financial or emotional support from their families during the lockdown. Having to spend time with unsupportive families was stressful for many MSM and in those times of difficulty, community friends extended mutual help through financial support as reported by a participant:

*“During the lockdown, I mostly talked to my [MSM] friends over the phone. I shared my financial issues and concerns over safety during COVID-19. I also helped my friends to buy rations … I was also vigilant about what I said over the phone while being at home. My family does not know about my sexual identity and HIV status.”* (IDI-7, *kothi*, age 35, married and living with family, HIV positive)

Virtual peer interactions through WhatsApp and Facebook served as key social support mechanisms following lockdown. Some participants formed WhatsApp groups consisting of close and trustworthy peers (including straight friends in some cases) to share their personal issues. A participant said,

*“I relieved myself from any mental stress during lockdown by talking to friends and cracking jokes with them. We, six to five close friends, created a WhatsApp group during the lockdown. It included my school friends, community friends, and straight friends.”* (IDI-8, *kothi*, age 25, single, sex worker)

These interactions afforded emotional and psychological support, with one participant reporting financial support from a peer. Virtual platforms also served as conduits for information, such as sites for free food distribution and financial support from NGOs and the government.

### Access to HIV and sexual health, and mental health services

Among those MSM who reported needing condoms during lockdown, about one-fifth (19%; *n* = 25/128) could not access them. Moreover, among those who sought HIV testing, 38% (*n* = 47/124) reported not being able to access it. Over half (55%; *n* = 65/119) of those who tried to see a doctor for STI screening or treatment were unable to do so, and three-quarters (74%; *n* = 48/65) could not access counselling services when needed.

In-depth interview findings revealed that fear of Coronavirus infection, diminished services from NGOs, and limited public transportation during lockdown hindered access to HIV/STI testing and treatment, and mental health services:

*“I did not do the HIV test as I was scared to go out. When I felt lonely or depressed, I shared it with my friends instead of meeting a [professional] counsellor. My friends in Delhi told me that their ART treatments were disrupted as they could not travel to access it*.*”* (IDI-1, gay, age 32, single)

### COVID-protective behaviours and condom use

Among those who reported sexual encounters since the beginning of lockdown (*n* = 132), 13% reported wearing a mask, one wearing gloves, 71% using hand sanitizers before sex, and 91% using condoms. Qualitative findings indicated some MSM involved in sex work were desperate for money and felt compelled to compromise protective measures. As reported by a participant: *“During the lockdown, I had to engage in sex without condoms as I was paid more. I know it is risky, but I needed money to buy food and pay the room rent”* (IDI-4, bottom, age 23, single). Other MSM engaged in sex work feared the loss of their already few clients during lockdown, and reported having to compromise their preferred condom use practices – though in this case, successfully maintaining condom use for anal sex: *“I refused kissing and condomless anal sex with any clients. As the client complained of less enjoyment, I had to compromise for oral sex without a condom”* (IDI-8, *kothi*, age 25, single, sex worker).

Sexual violence was also reported, with pandemic restrictions intensifying risk. For example, a sex worker recounted that his friend had been apprehended by police for curfew violation and forced to have sex with them:

*“I was scared to go out to find clients after hearing how my friend got harassed by the police [for curfew violation]. He was forcefully asked to have sex with the police, and we could not report it as he had not revealed his identity to his family.”* (IDI-4, bottom, 23 years, single, sex worker)

These narratives suggest that structural violence against MSM, particularly those of lower socioeconomic status (especially those in sex work) was exacerbated amid COVID-19 curfews and lockdowns.

## Discussion

The COVID-19 pandemic and public health responses have substantially impacted MSM in India both economically and psychologically and hindered access to health and mental health services, including HIV/STI prevention.^7^ Our study showed how COVID-19-related restrictions contributed to economic distress due to job layoffs, salary cuts and delays, and loss of income from sex work or daily wage labour, among an already economically marginalised population. These economic pressures, in turn, contributed to HIV risk and psychological distress.

The use of minority stress theory and syndemic theory offered insights into the interconnections between sexual minority status, health conditions and HIV risk among MSM during COVID-19. Our survey findings highlighted that both sexual minority stressors (internalised homonegativity) and COVID-19-specific stressors (social distancing) contributed to anxiety and depressive symptoms since the onset of lockdown measures. Qualitative findings revealed particular impacts of lack of supportive families and decreased access to friends and communities during the lockdown, which contributed to loneliness, depression, and anxiety. Well before the COVID-19 pandemic, MSM in India have been shown to face sexual minority-related stressors that contributed to psychological distress. Pervasive economic stressors during the COVID-19 pandemic have exacerbated their psychological distress.^12,13^

Qualitative findings further revealed intersectional vulnerabilities in the differential impact of stressors based on sex work involvement, living status, openness about sexuality, and family acceptance. For MSM living alone, loneliness and lack of peer support in discussing sexuality-related concerns further contributed to anxiety and depression. The higher odds of lockdown-related depression and anxiety among those with higher levels of internalised homonegativity is consistent with research demonstrating significant associations between internalised homonegativity and depression.^38^ For MSM living with families, constant vigilance in hiding one’s sexuality and inability to freely communicate with peers exacerbated psychological distress, similar to findings from pre-pandemic research.^39,40^ For MSM engaged in sex work, lack of clients and reduced income constrained condom use negotiation, increasing HIV risk, similar to the experiences of transgender women engaged in sex work in India.^6^ Lockdown conditions and economic hardship among sex workers were also exploited by clients and police, increasing risks of sexual violence and condomless sex.

Our findings are aligned with studies from other countries demonstrating high levels of psychological distress and HIV risk among LGBTQ communities during COVID-19.^33,41,42^ In India, social distancing and restricted access to public spaces similarly limit opportunities for social networking and peer interaction,^3,43^ increasing risks for depression and loneliness.^44^

Strengths of this study include the mixed methods approach, which provides initial evidence for the substantial impacts of the COVID-19 pandemic in India on mental health, economic well-being, access to health services and HIV risk among MSM, who are often not included in pandemic planning or responses.^8^ However, the results need to be understood in the context of study limitations. The use of convenience sampling precludes generalising findings to other MSM; nevertheless, the complementarity of findings across methods supports validity. The single-item outcome measures for lockdown-related depression and anxiety indicate the need for further research on pandemic-related psychological distress among MSM using standardised scales.

## Conclusion

The COVID-19 pandemic has resulted in considerable psychological and financial distress to low socioeconomic status MSM in India – communities already marginalised in economic, family and healthcare sectors. This includes MSM involved in sex work, with exacerbated risks of HIV transmission due to economic hardship and limited access to HIV preventive interventions. The study findings illustrate how COVID-19 has disproportionately and intersectionally impacted vulnerable communities in a lower-middle-income country like India and its implications for their sexual and mental health, and access to services. This study has highlighted the additional socioeconomic and health burdens faced by MSM during COVID-19 and their interconnections with sexual minority stressors.

Structural interventions such as ensuring inclusive health care policies, sensitisation of medical health providers, families, and the general public on sexual minority issues and economic empowerment through skill training are required to address the impact of COVID-19 on MSM. These interventions are crucial as MSM continue to experience structural inequalities in access to education, livelihood, and health care services. To a great extent, these structural inequalities are also shaped by stigma and discrimination towards sexual minorities. Our study has shown how these existing vulnerabilities are exacerbated in the context of COVID-19.

Based on the study findings, we recommend innovations in culturally competent programming and virtual service delivery to improve access to mental health support, including telehealth or virtual counselling, and access to HIV and STI services for MSM. This should include HIV self-testing, community-based testing, teleconsultations for pre-exposure prophylaxis (PrEP) and antiretroviral treatment, and support in navigating the combined risks of HIV and COVID-19.^45^ Interventions are also sorely needed to mitigate the financial burden of the pandemic, particularly for low socioeconomic status MSM, including day labourers and those engaged in sex work, who are often not eligible for government unemployment benefits, such as targeted low-interest loans and small grant support for unemployed MSM. Multisectoral programmes and policies designed with and for sexual and gender minority communiciates are crucial to address the multifaceted impacts of the COVID-19 pandemic and future pandemics and emergency situations.
